# *In vivo* detection of reduced Purkinje cell fibers with diffusion MRI tractography in children with autistic spectrum disorders

**DOI:** 10.3389/fnhum.2014.00110

**Published:** 2014-02-28

**Authors:** Jeong-Won Jeong, Vijay N. Tiwari, Michael E. Behen, Harry T. Chugani, Diane C. Chugani

**Affiliations:** ^1^Department of Pediatrics and Neurology, Wayne State UniversityDetroit, MI, USA; ^2^Translational Imaging Laboratory, PET center, Children's Hospital of MichiganDetroit, MI, USA

**Keywords:** Purkinje cell, dentate nucleus, autism spectrum disorders, diffusion weighted MRI, independent component analysis tractography with a ball and stick model

## Abstract

Postmortem neuropathology studies report reduced number and size of Purkinje cells (PC) in a majority of cerebellar specimens from persons diagnosed with autism spectrum disorders (ASD). We used diffusion weighted MRI tractography to investigate whether structural changes associated with reduced number and size of PC, could be detected *in vivo* by measuring streamlines connecting the posterior-lateral region of the cerebellar cortex to the dentate nucleus using an independent component analysis with a ball and stick model. Seed regions were identified in the cerebellar cortex, and streamlines were identified to two sorting regions, the dorsal dentate nucleus (DDN) and the ventral dentate nucleus (VDN), and probability of connection and measures of directional coherence for these streamlines were calculated. Tractography was performed in 14 typically developing children (TD) and 15 children with diagnoses of ASD. Decreased numbers of streamlines were found in the children with ASD in the pathway connecting cerebellar cortex to the right VDN (*p*-value = 0.015). Reduced fractional anisotropy (FA) values were observed in pathways connecting the cerebellar cortex to the right DDN (*p*-value = 0.008), the right VDN (*p*-value = 0.010) and left VDN (*p*-value = 0.020) in children with ASD compared to the TD group. In an analysis of single subjects, reduced FA in the pathway connecting cerebellar cortex to the right VDN was found in 73% of the children in the ASD group using a threshold of 3 standard errors of the TD group. The detection of diffusion changes in cerebellum may provide an *in vivo* biomarker of Purkinje cell pathology in children with ASD.

## Introduction

Autism spectrum disorders (ASD) are prevalent neurodevelopmental disorders characterized by impaired language development, repetitive or stereotyped behaviors, and difficulties in socio-emotional interactions (Kanner and Eisenberg, 1957; Fonbonne, 2003). Many neuroimaging studies demonstrate that the development of the cerebellum is abnormal in children with ASD, both neuroanatomically and functionally. For instance, abnormalities in cerebellar size, morphology, and function have been reported and correlated with behavioral deficits in functional domains (Abell et al., 1999; Courchesne et al., 2001; McAlonan et al., 2002; Akshoomoff et al., 2004; McAlonan et al., 2005; Fatemi et al., 2012). Neuropathology studies have shown significant reductions in Purkinje cells (PC) in the posterior-lateral cerebellar hemisphere in brain specimens from patients with ASD (Bauman and Kemper, 1985, 2005; Ritvo et al., 1986; Bailey et al., 1998; Whitney et al., 2009). In addition, decreased size of PC in autism brain specimens has been reported (Fatemi et al., 2002).

The PC are the primary efferent neurons of the cerebellar cortex. Loss of PC may result in altered cerebellar cortical efferent signals (Tsai et al., 2012) and may be associated with some of the symptoms that have been identified in children with ASDs including problems with motor control and learning (Hoxha et al., 2013). The dentate nuclei lie in a key position within the cerebellum, serving to integrate inputs from the PC efferents (Batini et al., 1992). Previous studies have reported no changes in cell number or size of the dentate nuclei in samples of children with ASDs (Bauman and Kemper, 1985; Yip et al., 2009). However, a recent diffusion weighted MRI (DW-MRI) study demonstrated alterations in white matter in the dentatorubrothalamic pathway in high and low functioning children with ASD (Jeong et al., 2012). This study divided the dentate nucleus into four subdivisions, dorso-rostal, dorso-caudal, ventro-rostal, and ventro-caudal, in order to investigate four different dentatorubrothalamic pathways associated with motor and non-motor domains (Küper et al., 2011). It was found that children with ASD had significant differences in fractional anisotropy (FA), axial diffusivity (AD), and radial diffusivity (RD) in the dentatorubrothalamic tracts originating in both the dorso-rostal and ventro-caudal portions of the dentate nucleus compared to a typically developing (TD) control group. These diffusion changes were highly correlated with deficits in daily living skills and communication, respectively (Jeong et al., 2012).

To date, no neuroimaging studies have investigated white matter connecting the cerebellar cortex to the dentate nucleus in children with ASD. This is due to several technical challenges involved in accurately defining these pathways, and in particular, the problem of crossing fibers. In order to address this problem, the present study applied a newly developed tractography method for DW-MRI termed the “independent component analysis with a ball and stick model” (ICA+BSM) (Jeong et al., 2013). This method was developed to resolve the orientation of multiple fiber bundles in clinical DW-MRI data and thereby increase the feasibility of detecting changes in efferent white matter in young children with autism, potentially related to decreases in number and size of PC neurons in cerebellar cortex shown postmortem. In addition to the crossing fiber problem, clinical MRI scans performed on children with ASD are performed under sedation, while MRI studies of TD children performed for research purposes are conducted without sedation due to ethical issues. Comparing data in which one group is sedated and the other is not may be result in between-group differences in movement and physiological artifacts (Walker et al., 2011, 2012) related to the effects of sedation, potentially confounding the identification of hypothesized differences in diffusion metrics between diagnostic groups (i.e., ASD vs. TD). In order to address the problems of motion and physiological artifacts, we assessed the magnitude of these artifacts in the cerebellum and corrected for them using iRESTORE (Chang et al., 2012).

We hypothesized that decreased number and size of the PC may result in reduced directional coherence and detection of streamlines connecting cerebellar cortex with dentate nuclei. Decreased PC cell number and size might also cause significant changes in other conventional DW-MRI metrics such as FA, AD, RD, streamline volume (SV), and streamline count (SC) (Song et al., 2002, 2005; Budde et al., 2009; Jones et al., 2013). Such changes in DW-MRI metrics may provide *in vivo* measures related to the previous pathology findings (Bauman and Kemper, 1985; Ritvo et al., 1986; Bailey et al., 1998; Bauman and Kemper, 2005; Whitney et al., 2009). This study assessed diffusion differences between children with ASD and TD children, while assessing potential artifacts associated with these measurements.

## Methods and materials

### Subjects

This study included 15 children with ASD (age: 6.2 ± 3.1 years, range: 3.6–13.3 years, 11 boys) and 14 TD children (TD, age: 6.8 ± 3.1, range = 4.0–14.0 years, 11 boys). These subjects are a subset of subjects included in a previous study of the dentatorubrothalamic pathway (Jeong et al., 2012). The children with ASD were referred from the Children's Hospital of Michigan Pediatric Neurology Clinic based upon a clinical diagnosis of autistic disorder, Asperger disorder, or pervasive developmental disorder not otherwise specified made by pediatric neurologists using the *Diagnostic and Statistical Manual of Mental Disorders, 4th edition*, criteria.

Inclusion and exclusion criterion for ASD were detailed in our previous study (Jeong et al., 2012). In brief, inclusion criteria for the study required that children with clinical diagnoses of ASD meet criteria for an autism spectrum disorder according to the ADI-R. In the present study 12 of the children met or exceeded the clinical cutoff on all three sections of the ADI-R [(a) Qualitative Abnormalities in Reciprocal Social Behavior, (b) Qualitative Abnormalities in Communication, and (c) Restricted, Repetitive, and Stereotyped Patterns of Behavior] and received diagnoses of Autistic Disorder. The remaining three children met or exceeded the cutoffs for criteria (a) and (c) and were diagnosed with Asperger's Disorder. Adaptive behavior was measured using the Vineland Adaptive Behavior Scales. The Vineland Adaptive Behavior Scales-2nd Edition (VABS) is a caregiver-reported semi-structured interview that yields measures of the child's adaptive behavior functioning in four domains (communication, daily living, socialization, and motor skills), as well as an overall adaptive behavior composite. The measure is used extensively in research studies on children with developmental disabilities and has excellent reliability and validity (Sparrow et al., 1984). Neurological disorders were excluded in the ASD group, including seizure disorders (patients with abnormal EEG without seizures were not excluded), PKU, tuberous sclerosis complex, Rett Syndrome, Fragile X, Down Syndrome and traumatic brain injury. The Human Investigation Committee at Wayne State University granted permission for the retrieval and analysis of the clinical data and MRI scans of children with ASD. Written informed consent was obtained for the children in the TD group.

### MRI data acquisition and processing

A 3T Signa EXCITE scanner (GE Healthcare, Waukesha, WI) equipped with an eight channel phased-array head coil was utilized to acquire the whole brain DW-MRI data at *TR*/*TE* = 12,500/88.7 ms, voxel size = 1.88 × 1.88 × 3 mm. A multislice single-shot echo-planer spin-echo sequence was employed to obtain the measurements at a diffusion weighting of *b* = 1000 s/mm^2^ and 55 diffusion gradient directions. An additional acquisition at *b* = 0 s/mm^2^ was also obtained to normalize the diffusion weighted signals at individual gradient directions. Parallel imaging of DW data acquired with the eight-channel EXCITE head coil was accomplished using the array spatial sensitivity encoding technique with an acceleration factor of 2. A three-dimensional fast spoiled gradient echo sequence (FSPGR) was acquired for each subject at TR/TE/TI of 9.12/3.66/400 ms, slice thickness of 1.2 mm, and planar resolution of 0.94 × 0.94 mm^2^. Since the participants in the ASD group underwent clinical scans, they were sedated during their scans. TD children were not sedated, but their movements were carefully monitored during the scans. In order to quantify head motion, an estimated head motion index (sum of displacements) was obtained from individual children in the TD and the ASD groups. For each child, *a*, *b* = 0 image was selected as a target image for co-registering the 55 *b* = 1000 images. Six motion parameters including three translation and three rotation parameters in *x,y,z* were estimated for each *b* = 1000 image using SPM 8 (http://www.fil.ion.ucl.ac.uk/spm/). For each parameter, the absolute displacement between adjacent images was averaged to assess the degree of head motion (Ling et al., 2012). The summation of the six motion assessments was used to denote the overall degree of head motion for each child.

We utilized a software package called the Tolerably Obsessive Registration and Tensor Optimization Indolent Software Ensemble (TORTOISE version 1.4.0. available from https://science.nichd.nih.gov/confluence/display/nihpd/TORTOISE) in order to (1) preprocess the DW-MRI data for correction of motion and eddy current distortion using DIFF_PREP, (2) estimate diffusion tensor data using informed Robust Estimation of Tensors by Outlier Rejection (iRESTORE) using DIFF_CALC, and (3) calculate the maps of FA, AD, and RD from the tensors of iRESTORE. The iRESTORE method utilizes an iterative non-linear least square fitting with equal weight to identify optimal outlier data on a voxel-by-voxel basis (Chang et al., 2012). It removes the identified data from consideration in the final tensor fitting, and performs conventional fitting on the remaining data points. It was designed to remove physiological noise artifacts and head motion in DW-MRI data acquired at low angular resolution.

### ICA+BSM tractography

The ICA+BSM tractography was performed using the TORTOISE-corrected DW-MRI data to identify the crossing fiber components in voxels of small clusters where dimensionality reduction and BSM fitting are sequentially applied to isolate the multiple diffusion components that are independently attenuated in each direction of the diffusion sensitizing gradients (Jeong et al., 2013). An eleven-neighborhood window was defined at each voxel of the white matter to create a diffusion data matrix with row vectors indicating the diffusion-weighted signals at every voxel of the window. Multiple diffusion tensors (up to 3) were estimated by iterating two complementary steps, hidden source decomposition using fast ICA and the multi-compartment ball-stick model.The resulting tensors were utilized to resolve the major fiber directions existing at the voxel and were finally applied for subsequent tractography. At each seeding point, tracking was started in the direction of the most prominent stick compartment. The step size was 0.2 voxels width, and the turning angle threshold was 60°. The propagation direction was calculated by applying trilinear interpolation on the directions of the stick compartments having a fraction >0.15, provided from 8 nearby voxels of the current point. For each nearby voxel, only the direction that had the smallest turning angle was considered for interpolation. In order to smooth the streamlines, each subsequent direction was determined by the previous direction with 0.5 weighting and the incoming direction with 0.5 weighting.

### Visualization of tracts connecting the cerebellar cortex with the dentate nuclei

To generate tracts containing the PC efferent fibers from the ICA+BSM tractography of individual subjects, the current study defined seeding points at the posterior-lateral region of the cerebellar cortex (e.g., cerebellum crus 1 and 2). The conventional FreeSurfer process (http://surfer.nmr.mgh.harvard.edu) was applied to the high resolution FSPGR images in order to segment the cerebellar cortex in each hemisphere. The resulting cerebellar cortex was then masked by the standard templates of cerebellum crus 1 and 2 (available at http://www.cyceron.fr/index.php/fr/plateforme/freeware). To seed the streamlines containing the PC efferent fibers, the masked region was finally registered to the b0 image via rigid body transformation using SPM 8 (http://www.fil.ion.ucl.ac.uk/spm/). A total of 2000 seeding points were uniformly distributed over all the voxels of the registered seed region.

**Figure 1:**
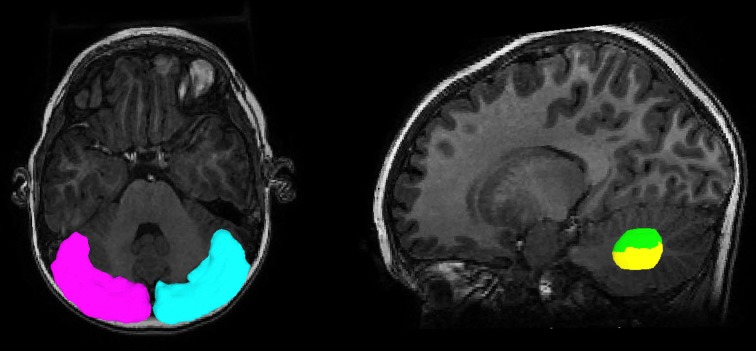
**Regions of interest to track streamlines containing PC efferent axons.** The posterior-lateral cortex of the cerebellum (seed region, magenta for left, cyan for right) and two subdivisions of the dentate nucleus (target region, green for DDN, yellow for VDN) were objectively placed for the tractography using the SUIT normalization procedure.

An ROI approach was utilized to sort the tracts connecting from the seed region to each of two dentate ROIs, the dorsal dentate nucleus (DDN) and the ventral dentate nucleus (VDN), which are considered to be the motor and non-motor domains of the dentate nucleus (Küper et al., 2011). The two subdivisions of the dentate nucleus in template space [using “Spatially Unbiased Infratentorial Template (SUIT)”] were separately transformed into the FSPGR space of the individual subjects by applying the inverse of the deformation field that fits the cerebellar cortex of the individual FSPGR image to that of the SUIT space (Diedrichsen, 2006, available at http://www.icn.ucl.ac.uk/motorcontrol/imaging/suit.htm). The SUIT normalized ROIs in the FSPGR space were registered to the b0 space by applying the rigid body transformation obtained between the FSPGR and b0 image using SPM 8 (http://www.fil.ion.ucl.ac.uk/spm/). Figure 1 illustrates an example of the cerebellar cortex seeding ROIs and the two dentate subregion sorting ROIs (DDN, VDN) that were objectively located in the b0 image. For each of the pathways projecting to the DDN and VDN, a streamline visitation map was created by the number of streamlines passing each voxel. Voxels having more than 5 visits were assumed to belong to each pathway, and the values of FA, AD, and RD for the voxels in each pathway was averaged for comparison. SV was measured by summing the volume of all voxels belonging to the pathway. SC was calculated by counting the total number streamlines per pathway. FA, AD, RD, SC, and SV were separately measured for each pathway bilaterally and compared to quantify diffusion metrics potentially associated with decreased PC size and number in children with ASD.

### Statistical analysis

Separate multi-variate general linear model analyses using four different dependent variables (left DDN, left VDN, right DDN, right VDN) were applied for each diffusion parameter to investigate differences between the TD and ASD groups. For these analyses, age and head-motion were used as covariates where head-motion was assessed for the individual subjects by summing the absolute differences of displacement estimates between adjacent *b* = 1000 images in six motion parameters, including three translations and three rotations (Ling et al., 2012). Finally, all diffusion parameters were correlated with developmental and behavioral variables (VABS assessments of motor, communication, daily living skills, and socialization) within the ASD group. For these analyses, age and head-motion were used as covariates, and partial Pearson correlation coefficients were obtained. SPSS 21.0 was used for the statistical analyses.

## Results

### Assessment of motion and physiological artifacts

**Figure 2:**
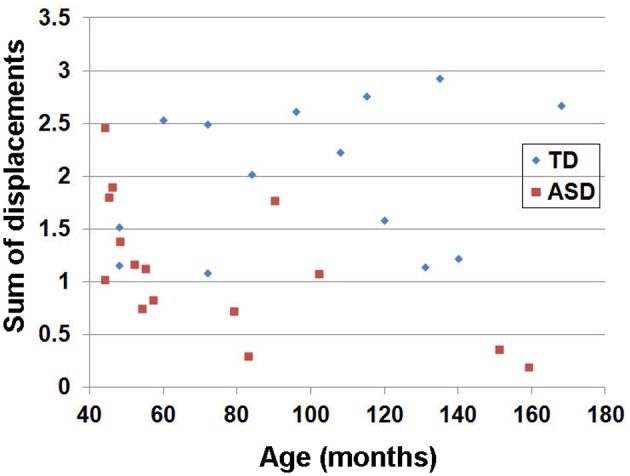
**Estimated head motion index (sum of displacements) obtained from individual children in the TD and the ASD groups.** For each child, *a*, *b* = 0 image was selected as a target image for co-registering the 55 *b* = 1000 images. Six motion parameters including three translation and three rotation parameters in *x,y,z* were estimated for each *b* = 1000 image. For each parameter, the absolute displacement between adjacent images was averaged to assess the degree of head motion (Ling et al., 2012). The summation of the six motion assessments was used to denote overall degree of head motion for each child. Note that this index has no unit since two physical metrics (mm and radian) are summed.

The estimated head motion index (sum of displacements) obtained from individual children in the TD and the ASD groups is shown in Figure 2. Head motion is higher in the TD group than in the ASD group, as expected due to sedation of the ASD group (group average ± standard deviation of the sum of motion displacements: 2.00 ± 0.67 for the TD group and 1.12 ± 0.63 for the ASD group). Analyses with iRESTORE identified more outlier data points in the TD group (9.1 ± 1.3%) than in the ASD group (7.2 ± 0.6%).

### Visualization of tracts connecting the cerebellar cortex with the dentate nuclei

**Figure 3:**
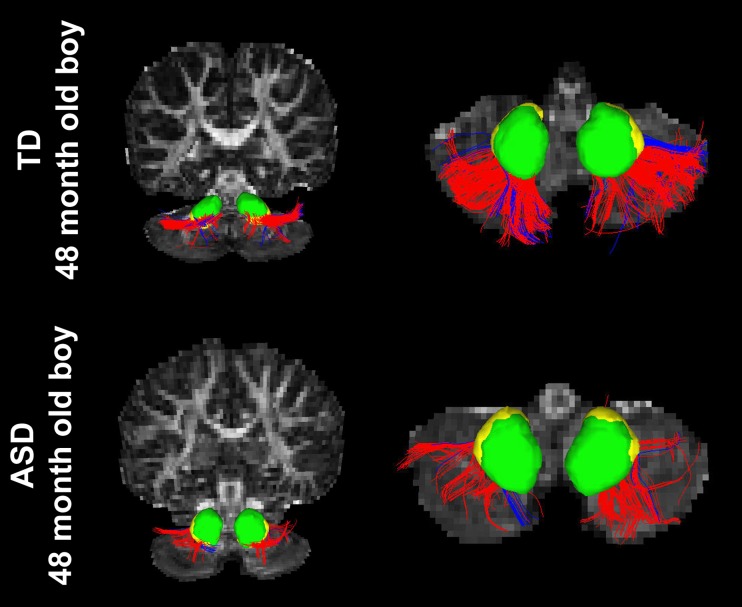
**Streamlines connecting the posterior-lateral cerebellar cortex with the DDN (red) and the VDN (blue) in two age-matched boys, (top) TD and (bottom) ASD.** It is visually apparent that the boy with ASD shows significantly reduced streamline number and volume compared to the TD child; total streamline volume of both sides = 13985 and 8933 mm^3^ for the TD child and the child with ASD, respectively.

**Figure 4:**
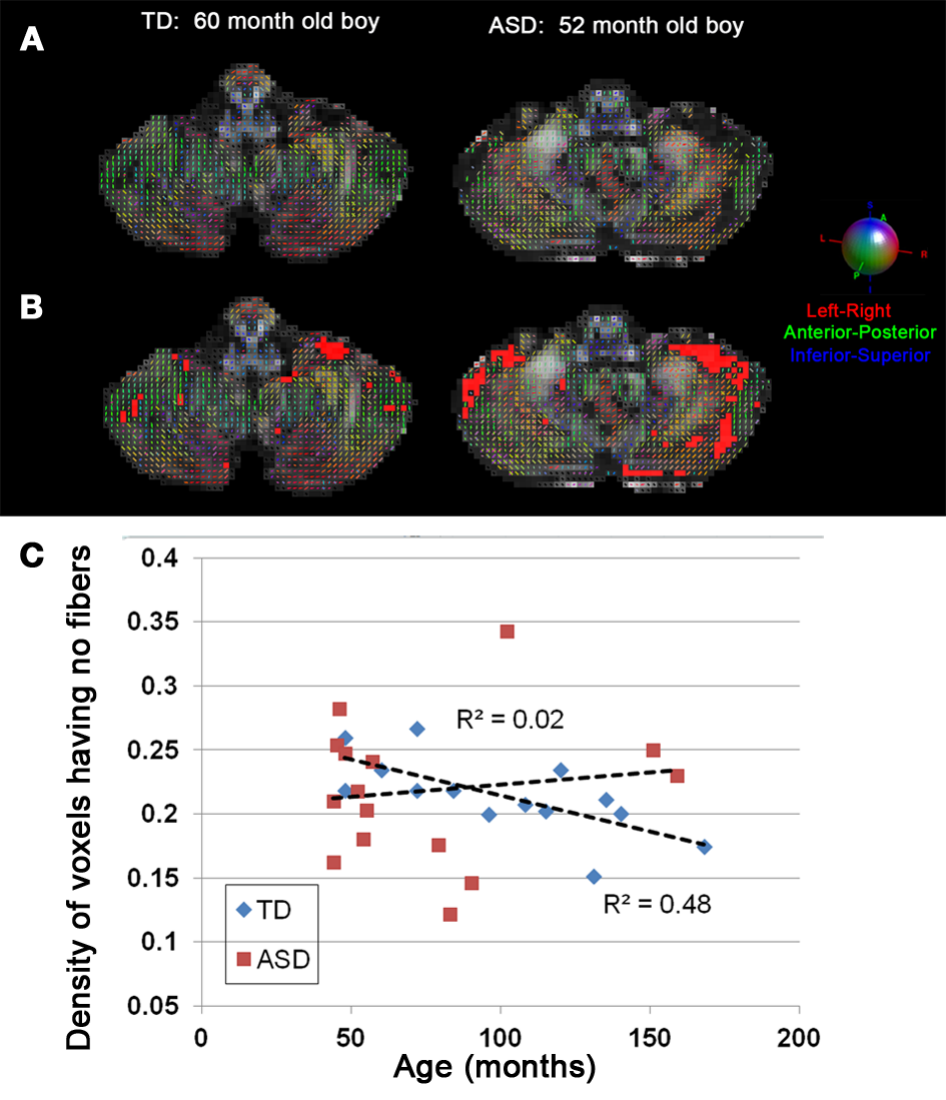
**(A)** Directions of stick compartments obtained by the ICA+BSM in two boys, (left) TD and (right) ASD. Each colored bar represents primary orientation of individual axonal bundle within the voxel of cerebellar white matter. **(B)** Voxels having no stick compartments were denoted by red boxes. **(C)** The density of cerebellar voxels having no stick compartments was evaluated in individual children in both the TD and ASD groups. To avoid a confound of size of the cerebellum, the total number of voxels having no stick compartments was normalized by total number of voxels in entire cerebellum which yields the density of voxels having no stick compartments. The TD group shows a significant age-related decrease in the density of voxels having no stick compartments (*R*^2^ = 0.48, *p* = 0.0044), while the ASD group shows no age related decrease (*R*^2^ = 0.02, *p* = 0.619).

Figure 3 shows representative examples of streamlines connecting the cerebellar cortex and the dentate nuclei in age-matched boys with TD and ASD. It is visually apparent in this figure that the SV in the posterio-lateral cerebellar cortex is reduced in the child with ASD, compared with the TD child. Although the decrease in streamlines was striking in some children with ASD as shown here, there was great variability among the children in the group. Both SC and SV were significantly lower only in the pathway projecting to the right VDN (*p* = 0.015 and 0.048 for SC and SV, respectively) in ASD group, compared to the TD group. Representative examples of directional compartments of streamlines identified by the ICA+BSM tractography (i.e., primary eigenvectors of the stick compartments) are shown in Figure 4A. The directional stick compartments are reduced near the voxels of the cerebellar cortex in the child with ASD (marked by red in Figure 4B), which may result in fewer streamlines in the child with ASD, compared with the TD child. Interestingly, age-related reduction of no stick voxels normalized by total cerebellum volume (i.e., density of voxels having no fibers) was notable in the TD group (*R*^2^ = 0.48, *p* = 0.0044) but not in the ASD group (*R*^2^ = 0.02, *p* = 0.619, Figure 4C).

### Comparison of diffusion parameters

**Figure 5:**
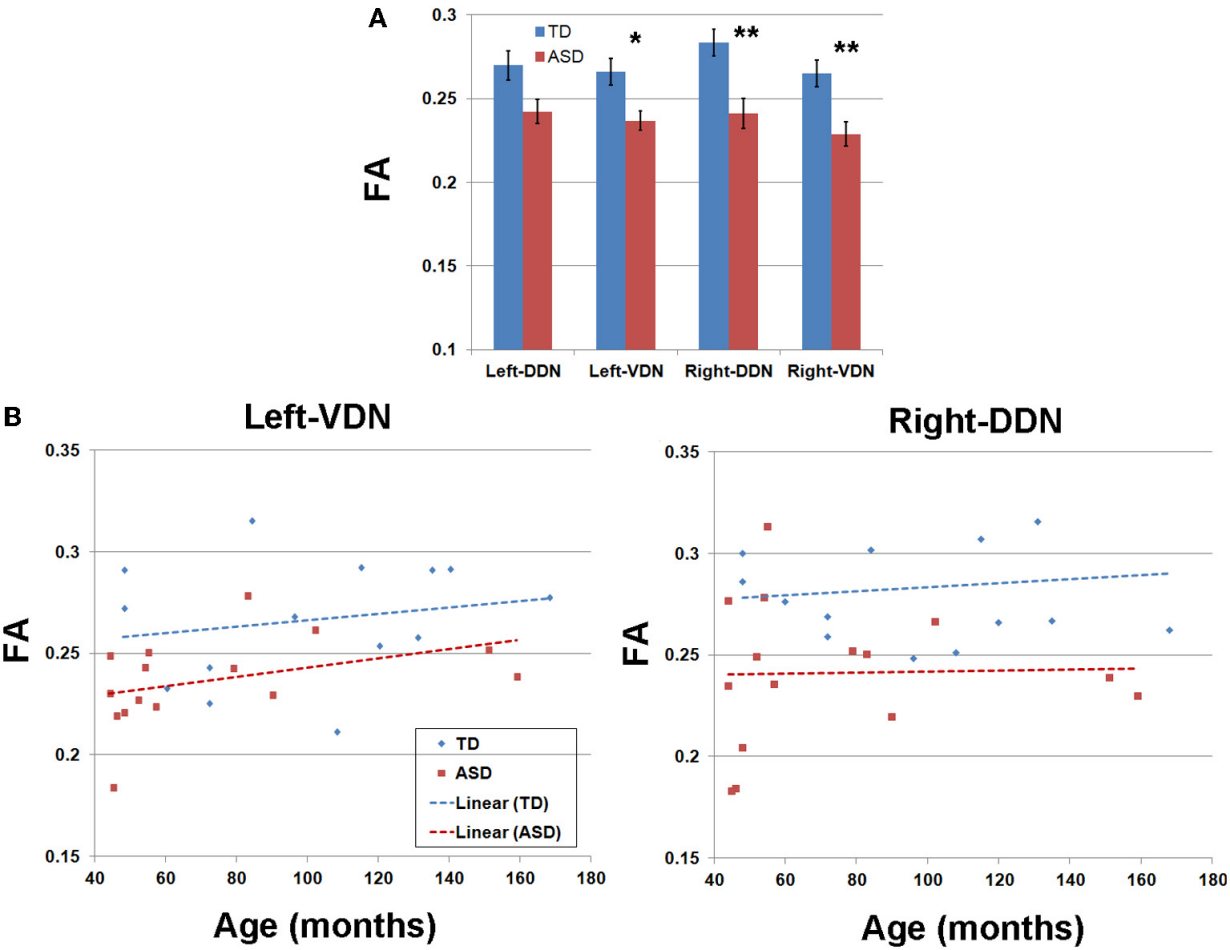
**(A)** Group mean values and standard errors of fractional anisotropy (FA) obtained from four pathways of the PC efferent streamlines. ^*^, ^**^p-value < 0.05 and 0.005, respectively (α = 0.05). **(B)** FA values and ages of individual subjects obtained from left VDN pathway (left) and right DDN pathway (right) showing the most significant group differences.

Tables 1, 2 summarize changes in four different diffusion parameters measured along pathways connecting the posterior-lateral cerebellar cortex with the dentate nuclei obtained from the TD and ASD groups. The mean values of the diffusion parameters showing significant group differences are given in Figure 5A. The multi-variate analyses revealed that FA was significantly lower in three pathways in the ASD group, compared to the TD group: pathways projecting to the right DDN (*p* = 0.008) and pathways projecting to the VDN bilaterally (left: *p* = 0.020, right: *p* = 0.010). In the left VDN and the right DDN, the reduced FA was apparent at all ages in children with ASD (Figure 5B). AD was significantly lower in the pathway projecting to the left DDN (*p* = 0.002) and to the left VDN (*p* = 0.040) in ASD group, compared to the TD group. However, RD was not significantly different in any of the pathways in ASD group compared to the TD group. Both SC and SV were significantly lower in the pathway projecting to right VDN in ASD group, compared to the TD group (*p* = 0.015, 0.048 for SC and SV, respectively). The FA difference between the ASD and TD groups for the right DDN pathway was more significant in older children (>5 y.o, Table 1), while the reduced AD in left DDN and VDN, and SV in the right VDN were more prominent in younger children (≤5 y.o, Tables 1, 2).

**Table 1:** **Fractional anisotropy (FA), axial diffusivity (AD), and radial diffusivity (RD) of the pathways connecting the posterior-lateral cerebellar cortex with the dentate nuclei**.

**Pathway**	**Group**	***FA***		***AD* [10^4^ mm/s^2^]**		***RD* [10^4^ mm/s^2^]**	
		**Mean ± *SD***	***p*-value**	**Mean ± *SD***	***p*-value**	**Mean ± *SD***	***p*-value**
Left DDN	TD	0.27 ± 0.03	0.083	8.97 ± 0.37	0.002^**^	5.76 ± 0.44	0.956
	ASD	0.24 ± 0.03		8.46 ± 0.38		5.85 ± 0.38	
	TD_≤5y.o_	0.26 ± 0.03	0.161	9.14 ± 0.23	0.000^**^	5.93 ± 0.57	0.640
	ASD_≤5y.o_	0.23 ± 0.03		8.53 ± 0.25		6.04 ± 0.33	
	TD_>5y.o_	0.28 ± 0.03	0.224	8.53 ± 0.25	0.054	5.66 ± 0.34	0.552
	ASD_>5y.o_	0.26 ± 0.02		8.36 ± 0.54		5.56 ± 0.28	
Left VDN	TD	0.27 ± 0.03	0.020^*^	9.13 ± 1.12	0.040^*^	6.03 ± 0.80	0.273
	ASD	0.24 ± 0.02		8.51 ± 0.78		5.90 ± 0.32	
	TD_≤5y.o_	0.25 ± 0.03	0.071	9.06 ± 0.47	0.094	6.02 ± 0.49	0.9556
	ASD_≤5y.o_	0.23 ± 0.03		8.60 ± 0.44		6.03 ± 0.29	
	TD_>5y.o_	0.27 ± 0.03	0.121	9.17 ± 1.40	0.219	6.04 ± 0.96	0.421
	ASD_>5y.o_	0.25 ± 0.02		8.39 ± 0.54		5.70 ± 0.26	
Right DDN	TD	0.28 ± 0.03	0.008^*^	9.06 ± 0.97	0.514	5.76 ± 1.00	0.396
	ASD	0.24 ± 0.04		8.84 ± 0.51		5.93 ± 0.44	
	TD_≤5y.o_	0.28 ± 0.02	0.092	9.15 ± 0.40	0.181	5.74 ± 0.43	0.505
	ASD_≤5y.o_	0.24 ± 0.04		8.87 ± 0.31		5.91 ± 0.44	
	TD_>5y.o_	0.29 ± 0.04	0.0161^*^	9.03 ± 1.20	0.673	5.78 ± 1.23	0.727
	ASD_>5y.o_	0.24 ± 0.02		9.03 ± 1.20		5.97 ± 0.48	
Right VDN	TD	0.27 ± 0.03	0.010^*^	9.03 ± 1.24	0.630	5.84 ± 1.29	0.316
	ASD	0.23 ± 0.03		8.80 ± 0.52		6.05 ± 0.59	
	TD_≤5y.o_	0.26 ± 0.02	0.083	9.10 ± 0.45	0.221	5.59 ± 0.25	0.1222
	ASD_≤5y.o_	0.23 ± 0.03		9.10 ± 0.45		5.94 ± 0.43	
	TD_>5y.o_	0.27 ± 0.03	0.030^*^	8.98 ± 1.55	0.780	5.99 ± 1.61	0.7667
	ASD_>5y.o_	0.23 ± 0.01		8.79 ± 0.72		6.20 ± 0.78	

DDN, dorsal dentate nucleus; VDN, ventral dentate nucleus; SD, standard deviation; Group mean and SD were reported for TD (n = 14), ASD (n = 15).

*, ** p-value < 0.05 and 0.005, respectively (α = 0.05).

**Table 2:** **Streamline count (SC) and streamline volume (SV) of the pathways connecting cerebellar cortex to the dentate nuclei**.

**Pathway**	**Group**	***SC***		***SV* [mm^3^]**	
		**Mean ± *SD***	***p*-value**	**Mean ± *SD***	***p*-value**
Left DDN	TD	328.86 ± 245.73	0.511	4822.93 ± 2483.78	0.776
	ASD	221.23 ± 143.57		3668.28 ± 1676.55	
	TD_≤5y.o_	226.40 ± 146.10	0.400	3896.02 ± 1735.3	0.593
	ASD_≤5y.o_	314.33 ± 195.65		4485.94 ± 2012.32	
	TD_>5y.o_	385.78 ± 277.92	0.189	5337.88 ± 2771.98	0.579
	ASD_>5y.o_	222.00 ± 85.46		4665.23 ± 870.83	
Left VDN	TD	401.64 ± 291.83	0.230	5258.36 ± 2660.71	0.333
	ASD	198.27 ± 134.62		3304.01 ± 1558.77	
	TD_≤5y.o_	307.40 ± 73.39	0.544	4665.23 ± 870.83	0.354
	ASD_≤5y.o_	253.00 ± 183.93		3932.81 ± 1777.67	
	TD_>5y.o_	454.00 ± 356.47	0.172	5517.18 ± 3259.20	0.431
	ASD_>5y.o_	234.17 ± 114.64		4389.26 ± 1033.49	
Right DDN	TD	430.93 ± 219.79	0.196	5604.90 ± 1962.33	0.416
	ASD	271.19 ± 153.14		4048.79 ± 1557.02	
	TD_≤5y.o_	360.40 ± 178.18	0.305	5174.30 ± 1688.04	0.446
	ASD_≤5y.o_	286.11 ± 85.31		4599.61 ± 1068.87	
	TD_>5y.o_	470.11 ± 240.39	0.389	5844.13 ± 2156.96	0.462
	ASD_>5y.o_	470.11 ± 240.39		4981.64 ± 2160.28	
Right VDN	TD	533.34 ± 247.84	0.015^*^	5990.62 ± 1967.36	0.048^*^
	ASD	279.38 ± 124.69		3769.70 ± 1376.23	
	TD_≤5y.o_	487.80 ± 212.29	0.064	6039.14 ± 752.26	0.017^*^
	ASD_≤5y.o_	305.33 ± 126.87		4348.83 ± 1223.67	
	TD_>5y.o_	558.78 ± 274.33	0.050	5963.66 ± 2450.37	0.215
	ASD_>5y.o_	296.17 ± 131.23		4510.55 ± 1424.99	

DDN, dorsal part of dentate nucleus; VDN, ventral part of dentate nucleus; SD, standard deviation; Group mean and SD were reported for TD (n = 14), ASD (n = 15).

* p-value < 0.005, respectively (α = 0.05).

**Figure 6:**
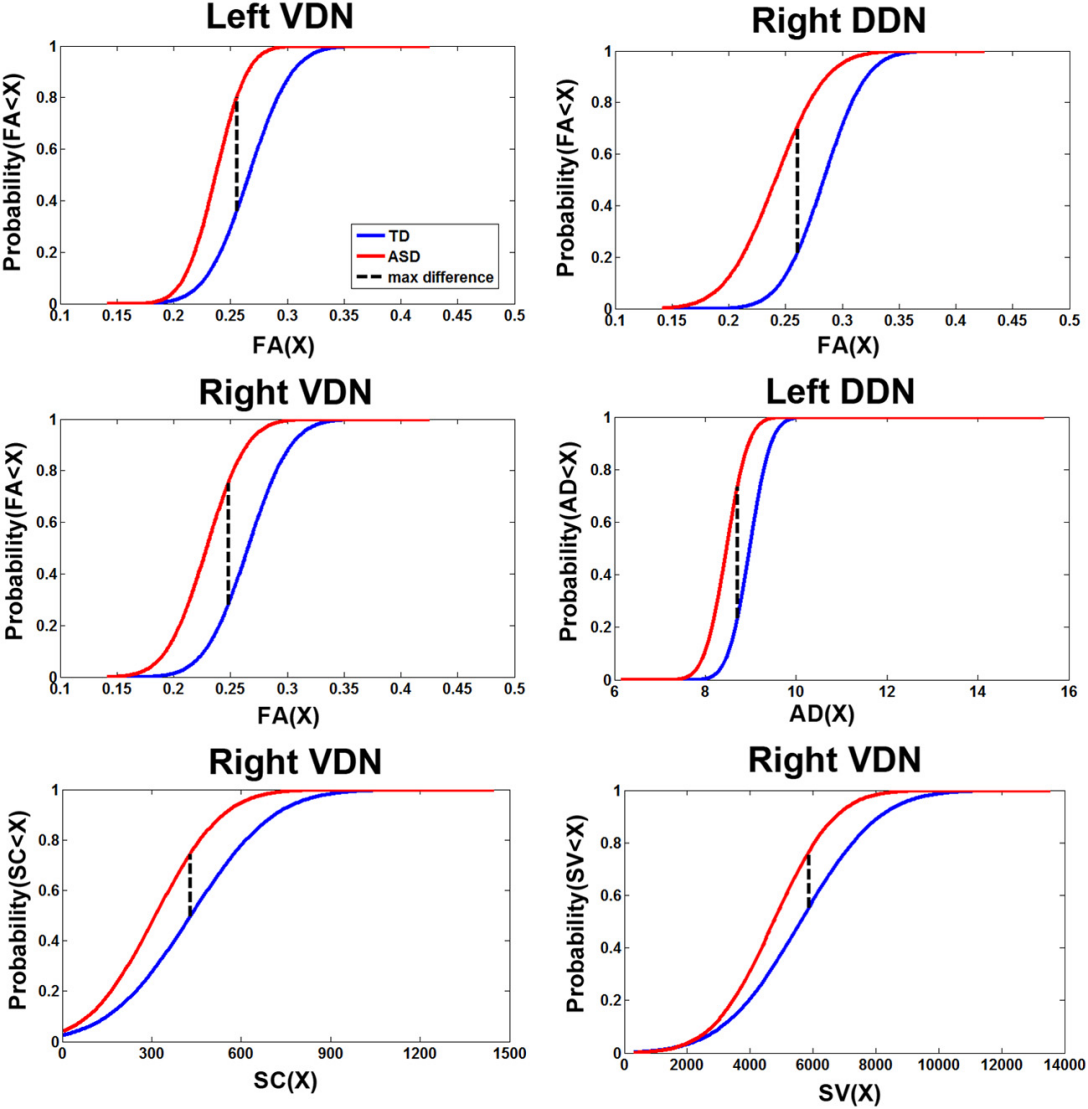
**Cumulative density functions of the diffusion parameters showing the significant group differences reported in Figure 5A.** Probabilities (parameter ≤X) were measured from the TD (*n* = 14) and ASD (*n* = 15) groups in the corresponding pathways showing significant group differences. The ASD group shows significantly higher probability to have a reduction in each parameter than the TD group in all pathways.

Figure 6 presents the cumulative density functions of FA, AD, SC, and SV measured from separate pathways showing significant changes between the TD and ASD groups. The ASD group shows significantly higher probability to have lower FA (left VDN, right DDN, right VDN), lower AD (left DDN, left VDN), lower SC (right VDN), and lower SV (right VDN) than the TD group, probably due to the reduced directional coherence of streamlines in ASD group. Furthermore, as reported in Table 3, FA was 3 standard errors below the mean of the TD group in 10 of 15 ASD cases (67%) for the right DDN pathway. For the right VDN pathway, a total 11 of the 15 ASD cases (73%) showed the reduced FA being 3 standard errors below the mean of the TD group.

**Table 3:** **Percentage of children with ASD showing significant changes in diffusivity parameters in the pathways connecting the cerebellar cortex with the dentate nuclei compared to the values of the TD group**.

**Pathway**	**Criterion**	***FA***	***AD***	***RD***	***SC***	***SV***
Left DDN	<lower 10% (TD)	27	53	0	0	0
	<Mean(TD)—3^*^SE(TD)	60	73	13	27	20
Left VDN	<lower 10% (TD)	33	0	0	0	7
	<Mean(TD)—3^*^SE(TD)	53	27	13	33	20
Right DDN	<lower 10% (TD)	53	0	0	0	7
	<Mean(TD)—3^*^SE(TD)	73	13	7	27	20
Right VDN	<lower 10% (TD)	47	0	0	7	0
	<Mean(TD)—3^*^SE(TD)	67	7	0	40	33

DDN, dorsal dentate nucleus; VDN, ventral dentate nucleus; SE, standard error; Group mean and SE were evaluated from TD (n = 14) and ASD (n = 15).

There was a trend for a positive correlation between FA of right DDN and daily living skills (*R* = 0.39, *p* = 0.084). There were no other correlations between diffusion parameters (AD, RD, SC, SV) and VABS variables (communication, socialization, and motor skills).

## Discussion

The present study demonstrates that the ICA+BSM tractography method can be used to detect differences in the cerebellar white matter that contain PC efferent pathways. Although the pathological origin of reduced directional coherence remains unclear in DW-MRI, the decreases in FA, AD, SC, and SV in the ASD group were hypothesized based upon previous pathology studies showing reduced numbers and size of PC in postmortem brain from subjects with autism (Bauman and Kemper, 1985, 2005; Ritvo et al., 1986; Bailey et al., 1998; Fatemi et al., 2002; Whitney et al., 2009). In addition to decreased numbers and size of PC in ASDs, there is also evidence for neuroinflammation throughout the brain in ASD; such inflammation is reported to be more prominent in cerebellum (Vargas et al., 2005; Suzuki et al., 2013). Changes associated with glial proliferation and inflammatory processes might be one source of the decreased directional coherence in children with ASD observed in the present study. We found that 73.3% of children with ASD (11 of 15 studied ASD cases, Table 3) showed reduced FA in fibers connecting cerebellar cortex to right VDN using a threshold 3 standard errors below the mean of the TD group. Similarly, Palmen et al. reported that 72.4% of subjects with ASD (21 of 29 studied cases) had a decreased number of PC (Palmen et al., 2004). Thus, the diffusion methods in the current study detected white matter pathology in pathways connecting the lateral cerebellar cortex to the dentate nuclei in a similar portion of cases as in postmortem pathology showing decreased PC in lateral posterio-lateral cerebellar cortex.

The mechanisms for the observed decreases in numbers of PC and other cerebellar pathology may reflect different etiologies. There is evidence for ASD genetic risk factors involving mutations in genes involved in cerebellar development such as Engrailed2 (Gharani et al., 2004) and the tuberous sclerosis genes TSC1 and TSC2 (Reith et al., 2011, 2013; Tsai et al., 2012), maternal infection and preterm birth (Pinto-Martin et al., 2011; Limperopoulos et al., 2014). Each mechanism might involve different aspects of PC development and maintenance. For example, PC might not be sufficiently generated, may fail to migrate to the proper layer or may degenerate later in development. There is evidence from human pathology studies that cells are formed, but are then lost. Bauman and Kemper (1994) hypothesized that that the PC loss occurred early in development at or before 30 weeks of gestation, associated with absence of glial cell hyperplasia and the preservation of neurons in the inferior olive. Whitney et al. (2009) suggested that PC are lost during the last trimester or early postnatal period potentially due to the preservation of basket and stellate cells in neuropathology samples from autistic subjects where there is PC loss. More recently, Wegiel et al. (2013) compared autism and control postmortem tissue and focused on the floccular region of cerebellar cortex. They reported focal areas of dysplasia in the flocculus with not only decreased PC, but also misaligned PC and loss of basket and stellate cells.

Mouse models in which the tuberous sclerosis genes TSC1 (Tsai et al., 2012) or TSC2 (Reith et al., 2013) are knocked down specifically in PC produced an increase in PC size followed by a progressive loss of PC. PC loss has been reported in two of four patients with TSC (Reith et al., 2011). Cerebellar abnormalities have also been shown following hypoxic or hypoxic-ischemic forebrain injury at postnatal day 2 in the neonatal rat (Biran et al., 2011). In this model, there were decreased numbers of PC and a decrease in the thickness of the molecular cell layer in multiple cerebellar lobules at postnatal day 21. The timing of the injury in this model would be similar to a very preterm infant born at 28 weeks gestation. An increased risk of ASD has been reported in preterm infants (Pinto-Martin et al., 2011) and infants with very low birthweight (Moss and Chugani, 2014). Furthermore, injury to the premature cerebellum in humans was significantly linked to autism (Limperopoulos et al., 2014). Preterm birth has also been associated with prenatal infection. Utilizing two mouse models, one involving maternal infection with the influenza virus, a second of maternal inflammation using poly I:C, Shi et al. (2009) found decreased numbers of PC and heterotopic PC in lobule VII, as well as delayed migration of granule cells in lobules VI and VII. Thus, there is evidence for multiple genetic and environmental risks for ASD that may lead to decreased PC number with or without changes in other cerebellar cell types and affecting different cerebellar cortical regions.

### Limitations of the study

Two common problems encountered in DW-MRI are (1) head motion and physiological artifacts (i.e., cardiac pulsation) and (2) the existence of multiple fiber orientations within an imaging voxel (referred to as the “intra-voxel crossing fiber problem”). A recent study reported that head motion resulted in a positive bias for the calculation of FA even though a standard correction method was applied to DW-MRI data (Ling et al., 2012). Indeed, it was also found that cardiac pulsation might influence the diffusion signals leading to over-estimation of the FA in cerebellum and underestimation of the FA in the genu and splenium of the corpus callosum in healthy adults (Walker et al., 2011). Since the ASD group was sedated and the TD group was not, there likely was more motion in the TD group. In addition, sedation of the ASD group may have systemically affected the heart rate and the breathing cycle resulting in altered patterns of physiological noise in the ASD group compared to the TD group. These types of artifacts were reported to be dominant in cerebellum (Walker et al., 2011, 2012).

To minimize the effects of these artifacts on the quality of the DW-MRI data, the present study utilized the iRESTORE tensor fitting to reduce variance and normalize the mean value of the metrics (Walker et al., 2011; Chang et al., 2012). iRESTORE fitting minimizes the variability of the metrics in the cerebellum by removing artifacts produced by both uncorrected head motion and the physiological noise including cardiac pulsation and respiration drop-out. Even though the iRESTORE tensor fitting was utilized to minimize the effect of physiological artifacts in both groups, the fitting algorithm might not correct the artifacts at all cerebellar voxels since the present study acquired the data without cardiac gating. In order to further correct for head motion, amount of head movement was included as a covariate in the statistical analyses. The group differences (i.e., ~10% reduction in FA) remained statistically significant even after covarying for age and head motion.

To address the intra-voxel crossing fiber problem, we utilized a novel tractography model called “ICA+BSM” which can provide the most accurate recovery of multiple streamlines in clinical DWI data, compared with other tractography methods (Jeong et al., 2013). Although the ICA+BSM was utilized to solve the problem of crossing fibers in the present study, it may not guarantee a complete solution for every voxel. In addition, the present study utilized SC as a measure of probable connection between the seeding region and the sorting ROI. Thus, the false estimates of PC efferent related streamlines may reflect uncertain change in geometry such as curvature, density, and length, suggesting that SC may be suboptimal to measure the degree of probable connection in PC efferent fibers (Jones et al., 2013). Further, the present study defined seeding points only in posterior-lateral cortex of cerebellum (see Figure 1) and therefore the methodology in the present this study would not detect PC pathology in other cerebellar cortical regions.

Additional limitations of the present study include the large age range of participants, the small sample size, the use of clinical diagnosis with ADI-R without a measure of direct observation (i.e., Autism Diagnostic Observation Schedule, ADOS) which is the current gold standard diagnostic instrument, and limited spatial-angular resolution of DWI data including the relatively small number of diffusion sensitizing gradients at a single b value. In addition, although the main connection between the cerebellar cortex and the dentate nuclei consists of PC efferent fibers, there is evidence of reciprocal afferents from the dentate to the cerebellum in several species (Brown and Graybiel, 1976; Tolbert et al., 1978). Finally, the apparent hemispheric asymmetry (more differences detected in pathways on the right side) in our study might be related to incomplete spatial normalization to identify cerebellar cortex and dentate nucleus in children with ASD. Although the present study successfully imaged changes in tracts containing PC efferent streamlines by combining conventional SUIT approach with FreeSurfer analysis, the current SUIT normalization scheme was reported to achieve about 94% of maximal overlap across participants and ±1.5 mm of registration error to locate the dentate nucleus in x-y-z axis (Diedrichsen et al., 2011). The error probably increases in younger children with ASD due to greater mismatch to the template. On the other hand, the right lateralized findings are consistent with other studies reporting differences in function in right and left cerebellum. For example, strongly right lateralized cerebellar intrinsic functional connectivity in the posterior lobe of cerebellum (crus I and II) with contralateral cerebral association cortex was reported in a study using resting state fMRI (Wang et al., 2013). Finally, functional asymmetries of tryptophan metabolism in cerebellum in children with ASD were also detected on α−^11^C-methyl-L-tryptophan positron emission tomography (Chugani et al., 1997; Eluvathingal et al., 2006).

## Conclusion

In summary, we used diffusion weighted MRI tractography to investigate whether structural abnormalities in cerebellar white matter (i.e., decreased PC numbers and size) that have been identified in postmortem specimens of individuals with ASD diagnoses could be detected *in vivo* in children with ASDs. Using this method we found reduced number and volume of streamlines from cerebellar cortex to dentate nuclei and reduced directional coherence in children with ASD diagnoses compared to TD children. Importantly, this method detected the white matter abnormalities at a similar proportion as has been reported in postmortem studies of ASD samples. Still, given the some of the limitations discussed above, these results are preliminary and further validation of this approach and replication of the above findings are warranted.

### Conflict of interest statement

The authors declare that the research was conducted in the absence of any commercial or financial relationships that could be construed as a potential conflict of interest.
